# Fully electric and plug-in hybrid cars - An analysis of learning rates, user costs, and costs for mitigating CO_2_ and air pollutant emissions

**DOI:** 10.1016/j.jclepro.2018.12.019

**Published:** 2019-03-01

**Authors:** Martin Weiss, Andreas Zerfass, Eckard Helmers

**Affiliations:** a European Commission, Joint Research Centre, Institute for Energy, Transport and Climate, Sustainable Transport Unit, via Fermi 2749, 21027, Ispra, Italy; b University of Applied Sciences Trier, Environmental Campus Birkenfeld, Environmental Planning and Technology Department, P.O. Box 1380, 55761, Birkenfeld, Germany

**Keywords:** Electric cars, Plug-in hybrid cars, Learning rates, Break-even production, Emissions mitigation costs

## Abstract

This article presents experience curves and cost-benefit analyses for electric and plug-in hybrid cars sold in Germany. We find that between 2010 and 2016, the prices and price differentials relative to conventional cars declined at learning rates of 23 ± 2% and 32 ± 2% for electric cars and 6 ± 1% and 37 ± 2% for plug-in hybrids. If trends persist, price beak-even with conventional cars may be reached after another 7 ± 1 million electric cars and 5 ± 1 million plug-in hybrids are produced. The user costs of electric and plug-in hybrid cars relative to their conventional counterparts are declining annually by 14% and 26%. Also the costs for mitigating CO_2_ and air pollutant emissions through the deployment of electrified cars tend to decline. However, at current levels, NO_X_ and particle emissions are still mitigated at lower costs by state-of-the-art after-treatment systems than through the electrification of powertrains. Overall, the observation of robust technological learning suggests policy makers should focus their support on non-cost market barriers for the electrification of road transport, addressing specifically the availability of recharging infrastructure.

## Highlights

• Since 2010, the price of electric and plug-in hybrid cars has been decreasing by 63% and 24%.
• Electric and plug-in hybrid cars show learning rates of 23 ± 2% and 6 ± 1%.
• Additional production of 7 ± 1 million electric cars and 5 ± 1 million plug-in hybrids before price break-even with conventional cars.
• Non-cost market barriers for electric vehicles such as recharging infrastructure demand more attention.

## 1 Introduction

Fully electric and plug-in hybrid cars have become increasingly popular, reaching market shares of 29% in Norway, 6% in the Netherlands, and 1.5% in China, France, and the UK (IEA, 2017). However, even a decade after their introduction into the mass-vehicle market, they continue to face important market barriers including high prices, short drive ranges, long recharging times, and an insufficient recharging infrastructure (Bonges and Lusk, 2016; Coffman et al., 2017; Gissler et al., 2016; Nilsson and Nykvist, 2016; FC, 2017; Liuima, 2017). The situation has been addressed, in part, by government incentives linked to ambitious deployment targets (IA-HEV, 2015; BR, 2016a; IEA, 2017). China and the USA, for example, aim at operating 5 million (SC, 2012) and 1.2 million electric vehicles (IA-HEV, 2015), respectively by 2020. Germany aims at having 1 million electric and plug-in hybrid cars on the roads by the same year (BR, 2016a). If these targets are to be achieved, persisting market barriers need to be removed by policy interventions that, in turn, require a good understanding of consumer preferences (Green et al., 2014) and techno-economic progress (IEA, 2016, IRENA, 2017a, IRENA, 2017b).

Specifically relevant to this context is technological learning - that is a decrease in production costs and improvements in product attributes through the combined effect of economies of scale, learning by doing, or learning by searching. Technological learning has been quantified for non-plug-in hybrid cars (Weiss et al., 2012a) and more recently for a small sample of electric cars (Safari, 2017). Both studies demonstrate a robust trend towards declining prices, implying that user costs and the costs for mitigating carbon dioxide (CO_2_) and air pollutant emissions through electrified vehicles may follow alongside. If so, electric and plug-in hybrid cars are not just becoming financially more attractive to consumers but also economically more efficient in mitigating the negative impacts of road transport (Helmers, 2010; Cames and Helmers, 2013; Degraeuwe et al., 2016).

This paper assesses the techno-economic performance of fully electric and plug-in hybrid cars sold in Germany - a country that constitutes the largest passenger car market in the EU with 3.4 million vehicle registrations in 2016 (KBA, 2016). The focus is on the time period between 2010 and 2016, for which we: (i) explore price trends and establish experience curves, (ii) conduct a time-series analysis of user costs, and (iii) assess the costs for mitigating CO_2_ and air pollutant emissions. The results will help policy makers to devise incentives that effectively support the electrification of road transport.

## 2 Methods

### 2.1 Definitions

Throughout this paper, we use the terms ‘electric car’, ‘fully electric car’, and ‘battery-electric vehicle (BEV)’ for passenger cars that are propelled by one or multiple electric motors and draw their propulsion energy solely from an electric battery. The terms ‘plug-in hybrids’, ‘plug-in hybrid car’, and ‘plug-in hybrid vehicle (PHEV)’ are used for passenger cars that: (i) are equipped with an internal combustion engine (ICE) and one or multiple electric motors, (ii) draw their propulsion energy from combustible fuels and/or electricity, and (iii) can be charged from an external electricity source. No distinction is made between *parallel* plug-in hybrids that can be propelled in parallel by the internal combustion engine and the electric motor(s) and *series* plug-in hybrids that are propelled by the electric motor(s) only. Our choice ensures a sufficiently large vehicle sample for the years 2011 and 2012 when only few plug-in hybrid car models were offered on the market. The terms ‘conventional car’ and ‘conventional vehicle (CV)’ are used for passenger cars propelled exclusively by an internal combustion engine that draws its energy from gasoline or diesel.

### 2.2 Data collection

We start by identifying through an extended web search all mass-produced electric and plug-in hybrid car models sold in Germany between 2010 and 2016, covering the period from their introduction into the mass-vehicle market to the point of writing. Electric cars whose traction battery is offered through a separate lease contract are excluded as these cars are cheaper than those sold with a traction battery (see Tables S1–S4 in the Supplementary Material).

For each identified electric and plug-in hybrid car model one comparable conventional car model was selected that matches its electrified counterpart, as far as feasible, in the production year, brand and model name, vehicle type and size, and engine power. We generally chose conventional cars with a manual transmission. The resulting bias is minor as the price difference between cars equipped with a manual transmission versus an automatic transmission is small, ranging between 300 and 1500 euro (EUR) per vehicle (ADAC, 2016).

For all identified electric, plug-in hybrid, and conventional car models information about the following parameters was collected: price [EUR], rated engine power [kW], if applicable the capacity of the traction battery [kWh], the certified distance-specific electricity or fuel consumption [kWh/100 km; l/100 km] and CO_2_ emissions [gCO_2_/km], and the certified emissions standard as published by car manufacturers or third-parties in print or online (see Tables S1–S4 in the Supplementary Material).

In a final step, relevant auxiliary information is collected. For calculating real vehicle prices, information about the value added tax is obtained from Statista (2017a) and information about the yearly inflation rate is obtained from Eurostat (2017). For estimating the cumulative production of electric cars and plug-in hybrids, data on the worldwide registration of new vehicles is obtained from ZSW (2016). For calculating user costs, we collected for each electric, plug-in hybrid, and conventional car model the costs of maintenance, insurance, and registration from ADAC (2017). Moreover, assumptions are made on vehicle lifetime, yearly mileage, real-world fuel and electricity consumption, and the price of diesel, gasoline, and electricity as indicated in Table 1.

**Table 1:** Data used for calculating user costs and the costs for mitigating CO_2_ and NO_X_ emissions through electric and plug-in hybrid cars (for further explanations see Table S5 in the Supplementary Material).

Parameter	Source	Electric cars	Plug-in hybrid cars	Conventional cars
Lifetime [years]	BMF (2017)	6/11^a^	6/11^a^	6/11^a^
Yearly mileage [km]	KBA (2015)	14,259	14,259	14,259
Electricity price [EUR/kWh]	BDEW (2017)	0.27	0.27	–
Fuel price [EUR_2015_/l]	Statista (2017b,c)	n.a.	1.31 (diesel) 1.49 (gasoline)	1.31 (diesel) 1.49 (gasoline)
Carbon intensity of the electricity mix [g CO_2_-equivalents/kWh]	Helmers et al. (2017)	707	n.a.	n.a.
Well-to-tank fuel losses [% of CO_2_ emissions at the tailpipe]	Fritsche (2007)	n.a	18	18
Difference between certified and real-world electricity and fuel consumption [% of certified value]	Zerfass (2015, 2017); Tietge et al. (2016)	30	218	year-specific estimates
NO_X_ emissions of power generation [g/kWh]	Helmers (2010)	0.44	0.44	n.a.
Carbon emissions of battery production [kg CO_2_ equivalents/kWh]	Moro and Helmers (2017)	168	168	n.a.

n.a. - not applicable.

a 11 years life time assumed in the sensitivity analysis.

The assumption of 6 years vehicle lifetime is motivated by three considerations: First, we ensure consistency with the collected data on maintenance costs (ADAC, 2017) that likewise refer to a lifetime of 6 years. Second, the assumption accounts for the uncertain lifetime of electric batteries (see, e.g., Helmers and Weiss, 2017; Myall et al., 2018) that is likely shorter than the life time of the car. Third, the assumption of 6 years vehicle lifetime is consistent with the depreciation period of 6 years as prescribed by BMF (2017) for commercially used cars in Germany. We acknowledge that passenger cars may be driven longer than for 6 years and consider in a sensitivity analysis an extended vehicle lifetime of 11 years (150,000 km). We do not account for battery replacement during this period and assume yearly maintenance and insurance costs are identical with those of cars operated within a lifetime of 6 years (see also discussion in Section 4.3.1).

The costs for mitigating CO_2_ and NO_X_ emissions by electric and plug-in hybrid cars were calculated based on gathered information as displayed in Table 1 and in Tables S2 and S4 in the Supplementary Material. Table 1 accounts for:

• certified and real-world electricity consumption of electric car models;
• certified and real-word CO_2_ emissions at the tailpipe of plug-in hybrid and conventional car models;
• CO_2_ emissions of electricity production in Germany;
• CO_2_ emissions of battery production.

To model in a sensitivity analysis the diffusion of renewables in the electricity mix, a carbon intensity of 131 g CO_2_-equivalents/kWh was assumed, which comprises the residual carbon emissions of largely renewable-based electricity (Helmers et al., 2017).

For calculating the cost of mitigating air pollutant emissions, the focus was on nitrogen oxides (NO_X_) and particle number (PN) emissions as both pollutants cause major concerns for public health (EEA, 2016a; WHO, 2016). We choose to address particle number emissions instead of particulate mass emissions as the former parameter captures more accurately the health effects of particles (Hennig et al., 2018), specifically those of ultrafine particles in the size range between >23 nm and 100 nm that contribute little to the overall mass of emitted particles (Mamakos et al., 2012). The following data were collected:

• distance-specific on-road NO_X_ and particle number emission factors for plug-in hybrid and conventional car models (Table 2);

**Table 2:** Tailpipe NO_X_ and particle number emission factors of plug-in hybrid and conventional cars; principal data sources: EEA (2016b), Giechaskiel et al. (2015), Hammer et al. (2015) (for further explanations see Table S6 in the Supplementary Material).

Pollutant	NO_X_ [mg/km]	Particle number [#/km]
Plug-in hybrid cars - Gasoline (Euro 5)	13	8 × 10^11^
Plug-in hybrid cars - Gasoline (Euro 6b)	13	3 × 10^12^
Plug-in hybrid cars - Diesel (Euro 5)	490	8 × 10^11^
Plug-in hybrid cars - Diesel (Euro 6b)	490	8 × 10^11^
Conventional cars - Diesel (Euro 5)	610	4 × 10^11^
Conventional cars - Diesel (Euro 6b)	500	4 × 10^11^
Conventional cars - Gasoline (Euro 5)	60	1 × 10^12^
Conventional cars - Gasoline (Euro 6b)	60	4 × 10^12^
• NO_X_ emissions of electricity production in Germany (Table 1).

The assumed NO_X_ emission factors are primarily based on the European Environmental Agency's air pollutant emission inventory guide book (EEA, 2016b, Table 2). The emission factors are within the range of values identified by on-road testing with Portable Emissions Measurement Systems (Weiss et al., 2012b; Yang et al., 2015). Data about the on-road NO_X_ emissions of plug-in hybrid cars are still scarce. Here, we rely on Franco et al. (2016) who conducted, to our knowledge, the only openly available on-road NO_X_ emission measurements of plug-in hybrid diesel cars.

On-road measurements of particle number emissions have only recently become available. The emission factors applied in this analysis are based on tests conducted on the chassis dynamometer and on the road (Giechaskiel et al., 2015; Hammer et al., 2015). Separate emission factors for gasoline and diesel car models as well as for models certified according to the Euro 5 and 6 emission limits were assumed. Thereby, the Euro 5 limit applies to cars sold between 2010 and 2014 whereas the Euro 6 limit applies to cars sold in 2015 and 2016 (Table 2).

### 2.3 Data analysis

#### 2.3.1 Data aggregation

The deflated and tax corrected real price *P*_*it*_ [EUR_2015_], referenced to the year 2015, was calculated for each electric, plug-in hybrid, and conventional car model as:

(1) $$P_{it}=\frac{p_{it}}{\left(1+r_{t}\right)k_{t}}$$

where *p*_*it*_ represents the nominal price of car model *i* in year *t* [EUR], *r*_*t*_ represents the value added tax rate in year *t*, and *k*_*t*_ represents the year-specific currency deflator which was calculated based on the yearly inflation rate. Afterward, the specific price [EUR_2015_/kW; EUR_2015_/kWh] of each car model was calculated by normalizing the real price *P*_*it*_ with: (i) the rated power [kW] and (ii) the battery capacity [kWh] in the case of electric cars.

In a final step, the price differential Δ*P*_*it*_ [EUR_2015_/kW] between each electric and plug-in hybrid car model *i* in year *t* and its conventional counterpart was calculated as:

(2) $$\Delta P_{it}=PE_{it}-PC_{it}$$

where *PE*_*it*_ represents the specific price of the electric and plug-in hybrid car model [EUR_2015_/kW] and *PC*_*it*_ represents the specific price of the comparable conventional car model [EUR_2015_/kW]. The real specific prices and price differentials were used in the first part of our analysis to explore price trends and establish experience curves.

#### 2.3.2 Experience curve analysis

Experience curves were established with SigmaPlot^®^ by plotting the yearly mean price [EUR_2015_/kW; EUR_2015_/kWh] and price differential [EUR_2015_/kW], denoted here as *P*_*t*_*(x*_*t*_*)*, for electric and plug-in hybrid cars as a function of cumulative vehicle production. Plotting the mean values instead of the individual prices and price differentials of all electric and plug-in hybrid cars ensures each year receives the same weight in the experience curve analysis. Then, a non-linear regression analysis was conducted by fitting the following power-law function to the data:

(3) $$P_{t}\left(x_{t}\right)=P_{0}\left(x_{0}\right){\left(\frac{x_{t}}{x_{0}}\right)}^{b}$$

where *P*_*0*_*(x*_*0*_*)* represents the mean price or price differential of electric and plug-in hybrid cars in the base years 2010 (electric cars) and 2011 (plug-in hybrids); *x*_*0*_ and *x*_*t*_ represent the cumulative production in the base year and in year *t* of the analysis; *b* represents the experience index, depicting the rate at which prices and price differentials of electric and plug-in hybrid cars decline. Depicting the resulting experience curve on a double-logarithmic scale yields a linear regression line with slope *b*. From this slope, the learning rate *LR* [%] was deduced as the rate at which prices and price differentials decline with each doubling of cumulative vehicle production:

(4) $$LR=\left(1-2^{b}\right)\cdot 100\%$$

The standard error of the slope parameter *b* obtained from Equation (3) is used to derive the error interval of learning rates. Equation (3) was also used to calculate the marginal cumulative production *x*_*BE*_ of electric and plug-in hybrid cars that is necessary to achieve a price break-even with conventional cars:

(5) $$x_{BE}=\sqrt[{b}]{\frac{P_{BE}}{P_{2016}}}x_{2016}$$

where *x*_*2016*_ represents the cumulative production in year 2016, *P*_*BE*_ the break-even price equal to the average price [EUR_2015_/kW] of conventional cars in 2016, and *P*_*2016*_ the average price of electric and plug-in hybrid cars, respectively in 2016. The error interval of the marginal cumulative production was estimated from the standard error of the experience index *b*.

#### 2.3.3 Time-series analysis of user costs

In the second part of the analysis, user costs *C*_*i,t*_ [EUR_2015_/km] of each electric, plug-in hybrid, and conventional car model *i* sold in year *t* were calculated as:

(6) $$C_{it}=\frac{P_{it}+\left(CM_{i}+M_{i}F_{i}CF\cdot 0.01\right)L_{i}}{M_{i}L_{i}}$$

where *P*_*it*_ represents the real absolute vehicle price [EUR_2015_], *CM*_*i*_ the yearly maintenance costs [EUR_2015_] comprising vehicle maintenance, registration, and insurance, *M*_*i*_ the yearly driving distance [km], *F*_*i*_ the distance-specific electricity or fuel consumption [kWh/100 km; I/100 km] under real-world conditions, *CF* the price of electricity or fuel [EUR_2015_/kWh; EUR_2015_/l], and *L*_*i*_ the lifetime [a] of each respective model *i*. For each year, the mean and standard deviation of user costs for all car models were calculated.

#### 2.3.4 Time-series analysis of costs for mitigating emissions

In the third part of the analysis, the costs for mitigating CO_2_ and air pollutant emissions *CE*_*it*_ [EUR_2015_/t CO_2_; EUR_2015_/t NO_X_; EUR_2015_/10^17^ particles] of each electric and plug-in hybrid car model *i* sold in year *t* were calculated as:

(7) $$CE_{it}=\frac{C_{it}\left(BEV-PHEV\right)-C_{it}\left(CV\right)}{E_{it}\left(CV\right)-E_{it}\left(BEV-PHEV\right)}$$

where *C*_*it*_*(BEV-PHEV)* represents the user costs of each electric and plug-in hybrid car model [EUR_2015_/km], respectively, *C*_*it*_*(CV)* stands for the user costs of the equivalent conventional car model [EUR_2015_/km], *E*_*it*_*(BEV-PHEV)* represents the distance-specific emissions of each electric and plug-in hybrid car model, and *E*_*it*_*(CV)* represents the distance-specific emissions of each conventional car model, respectively. The so-calculated costs *CE*_*it*_ represent the marginal costs of mitigating CO_2_ and air pollutant emissions below the emission levels of conventional cars. Costs can assume extremely large positive or negative values depending on the differences in user costs and emissions between electric and plug-in hybrid cars on one hand and their conventional counterparts on the other hand. Therefore, the calculated costs *CE*_*it*_ have to be interpreted with caution and after careful inspection of the underlying data. To avoid that outliers bias the cost estimates, we chose the median and half of the interquartile range to represent the general trend and variability in the costs for mitigating emissions through electric and plug-in hybrid cars.

The costs for mitigating CO_2_ emissions were calculated for four scenarios that consider: (i) the distance-specific tailpipe CO_2_ emissions as certified during type approval, (ii) the distance-specific tailpipe CO_2_ emissions under real-word driving conditions based on ICCT (2017), (iii) the distance-specific CO_2_ emissions along the entire well-to-wheel (WTW) electricity and fuel supply chain (see Table 1), and (iv) a hybrid WTW scenario proposed by Moro and Helmers (2017) that also includes the CO_2_ emissions from battery manufacturing (see Table 1). The latter scenario is justified as electric cars and conventional cars are composed of a largely comparable materials cake with the exception of the traction battery whose production is energy intensive (Moro and Helmers, 2017).

The costs for mitigating NO_X_ and particle emissions were calculated by considering: (i) the distance specific tailpipe NO_X_ and particle number emission under real-world driving conditions and (ii) in the case of NO_X_ additionally the emissions from electricity generation, accounting thereby for the indirect NO_X_ emissions caused by electric and plug-in hybrid cars (see Table 1).

## 3 Results

### 3.1 Price trends and experience curves

**Fig. 1:**
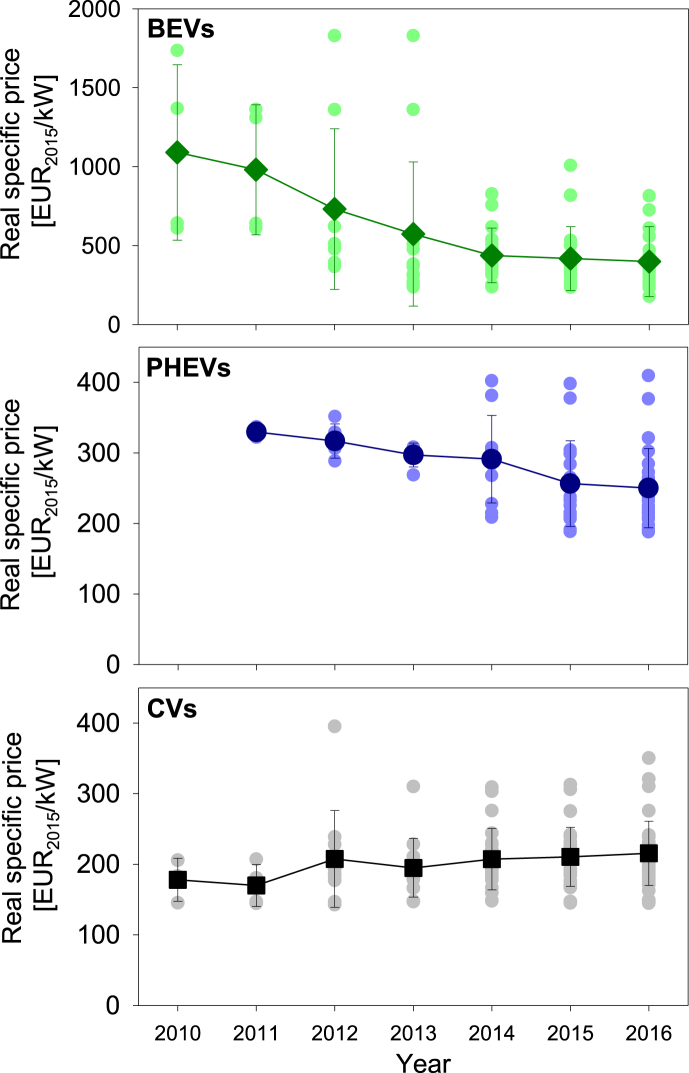
Specific price of electric cars (BEVs), plug-in hybrids (PHEVs), and conventional cars (CVs) sold in Germany; squares depict mean prices; error intervals represent the standard deviation of price data.

The mean price of electric cars sold in Germany has decreased by 63% from 1090 ± 560 EUR_2015_/kW in 2010 to 400 ± 220 EUR_2015_/kW in 2016; the mean price of plug-in hybrids has decreased by 24% from 330 ± 10 EUR_2015_/kW in 2011 to 250 ± 60 EUR_2015_/kW in 2016. By contrast, the mean price of comparable conventional cars has increased by 21% from 180 ± 30 EUR_2015_/kW in 2010 to 220 ± 50 EUR_2015_/kW in 2016 (Fig. 1).

**Fig. 2:**
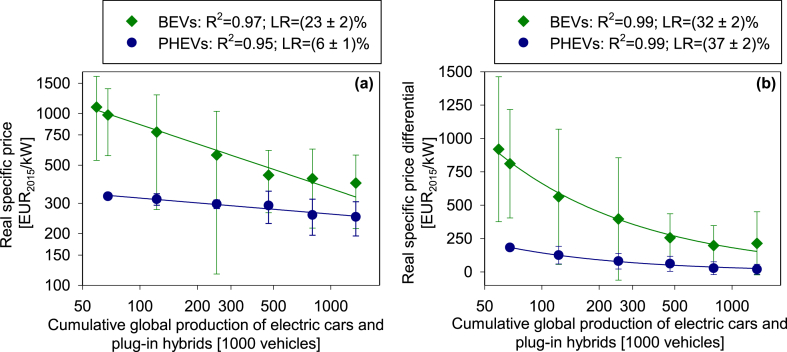
Experience curves depicting the mean specific price (a) and the mean specific price differential (b) of electric cars and plug-in hybrids; error intervals represent the standard deviation of data.

The prices of individual models scatter over a wide range. Although electric cars still tend to be on average more expensive than their conventional counterparts, the robust price decline suggests substantial technological learning in the electrification of powertrains. In fact, the experience curve analysis reveals learning rates of 23 ± 2% and 6 ± 1% for the specific price of electric cars and plug-in hybrids, respectively (Fig. 2a). Even higher learning rates of 32 ± 2% and 37 ± 2% are observed for the price differential between electric cars and plug-in hybrids and their conventional counterparts (Fig. 2b).

The mean price differential between electric and conventional cars has decreased from 920 ± 540 EUR_2015_/kW in 2010 to 214 ± 237 EUR_2015_/kW in 2016. The mean price differential between plug-in hybrid and conventional cars has decreased from 182 ± 11 EUR_2015_/kW in 2011 to 20 ± 38 EUR_2015_/kW in 2016, suggesting plug-in hybrids are close to price parity with comparable conventional cars. Expressing the price of electric cars in terms of battery capacity yields a learning rate of 16 ± 2% (see Text Box 1 in the Supplementary Material).

Assuming (i) the learning rates for electric cars and plug-in hybrids apply in the future and (ii) the prices of conventional cars remain as in 2016, an additional 7 ± 1 million electric cars and 5 ± 1 plug-in hybrids have to be produced before reaching price break-even with conventional cars. These numbers are remarkably low and account for less than 10% of the annual global production of passenger cars (OICA, 2018).

### 3.2 Time-series of user costs

**Fig. 3:**
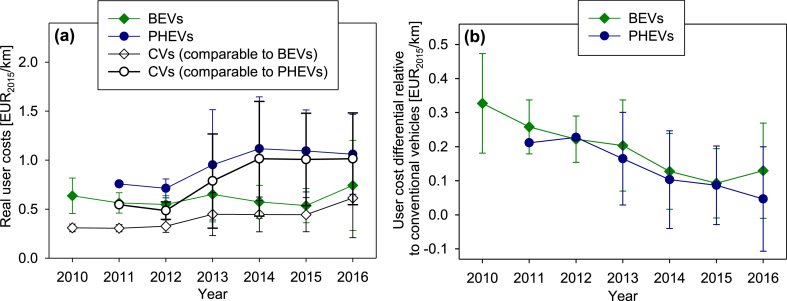
Mean user costs (a) of electric cars (BEVs), plug-in hybrids (PHEVs), and conventional cars (CVs) and mean differential user costs of electric cars and plug-in hybrids relative to conventional cars (b); error intervals represent the standard deviation of data.

User costs do not follow the trend of vehicle prices but tend to remain constant (electric cars) or increase (plug-in hybrids and conventional cars) between 2010 and 2016 (Fig. 3a). This observation suggests that the decline in specific vehicle prices is compensated by a trend towards more powerful vehicles and subsequently an increase in absolute vehicle prices as well as the electricity and fuel consumption of vehicles (Zerfass, 2017). In 2016, electric cars, plug-in hybrids, and their conventional counterparts cost their users 0.74 ± 0.46 EUR_2015_/km, 1.06 ± 0.41 EUR_2015_/km, and 0.71 ± 0.44 EUR_2015_/km, respectively. The latter number represents the user costs of all conventional cars contained in our analysis.

The user costs of electric cars, plug-in hybrids, and their conventional counterparts decrease to 0.51 ± 0.30 EUR_2015_/km, 0.75 ± 0.27 EUR_2015_/km, and 0.52 ± 0.29 EUR_2015_/km in 2016 when considering an extended vehicle life time of 11 years and 150,000 km (Table S7 in the Supplementary Material).

The high user costs of plug-in hybrids relative to electric cars can be attributed to their high absolute price, power, and electricity/fuel consumption. The differential user costs of electric cars and plug-in hybrids compared to conventional cars have been declining in the period of analysis by 60% and 78%, which translates into an annual decline of 14% and 26%, respectively. By 2016, electric cars and plug-in hybrids cost their users 0.13 ± 0.14 EUR_2015_/km and 0.05 ± 0.15 EUR_2015_/km more than conventional cars do (Fig. 3b), suggesting that the former cannot recover, on average, their price premium within a lifetime of 6 years. However, when assuming a life time of 11 years, electric cars and plug-in hybrids are cost effective already to date. In this scenario, individual electric cars and plug-in hybrids in fact can cost their users less than conventional cars whereas on average additional user costs scatter around 0.05 ± 0.09 EUR_2015_/km for electric cars and 0.02 ± 0.11 EUR_2015_/km for plug-in hybrids (see also Table S7 in the Supplementary Material).

### 3.3 Time-series of emissions mitigation costs

#### 3.3.1 Costs of mitigating carbon dioxide emissions

**Fig. 4:**
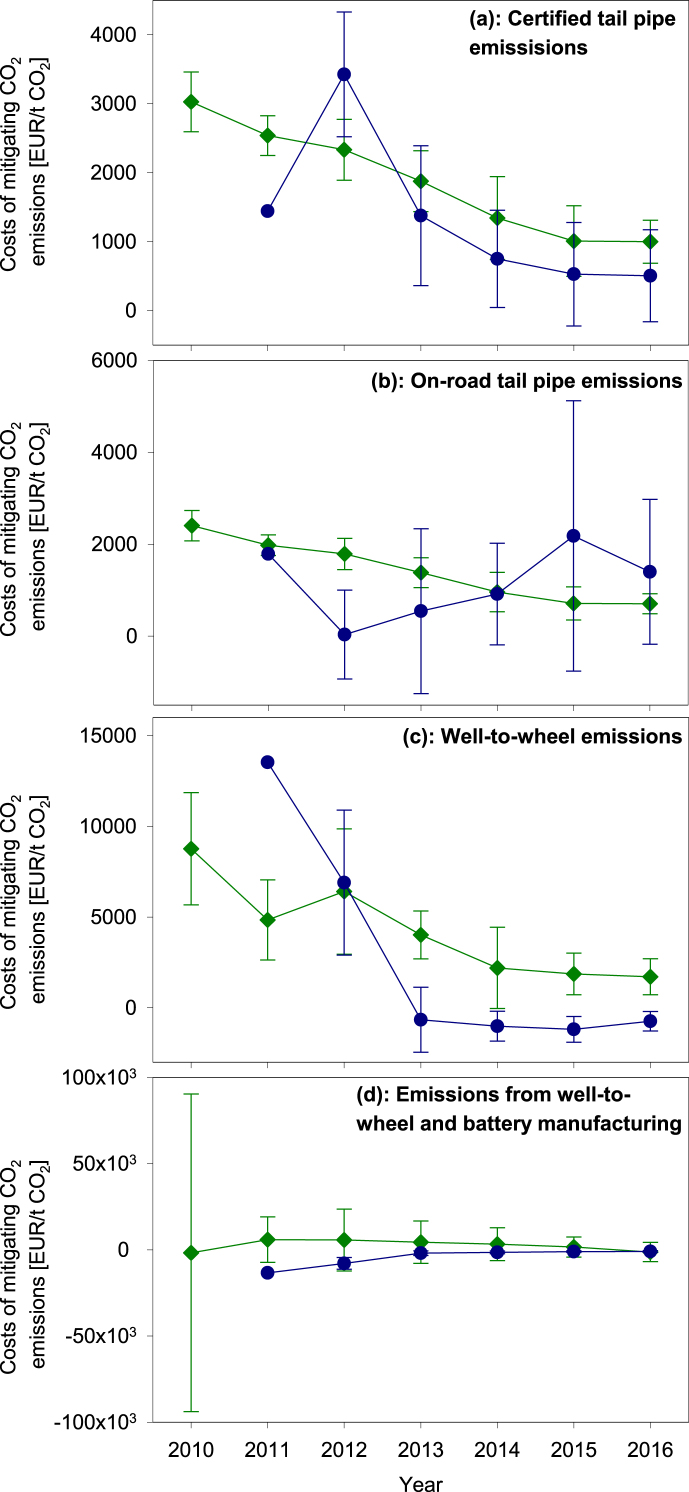
Median costs for mitigating CO_2_ emissions of conventional cars by electric cars (green diamonds) and plug-in hybrids (blue circles) considering certified tailpipe emissions (a), on-road tailpipe emissions (b), emissions along the entire well-to-wheel chain of electricity and fuels (c), and a hybrid approach including well-to-wheel emissions and those from battery production (d); error intervals represent half of the interquartile range of cost data; a sample size of one model does not permit presenting an error interval for plug-in hybrid cars in 2011.

The CO_2_ emissions of electric, plug-in hybrid, and conventional cars vary depending on the scenario considered. Thus, also the costs for mitigating the CO_2_ emissions of conventional cars through the deployment of electric cars and plug-in hybrids are scenario dependent. Fig. 4 suggests that:

• The CO_2_ mitigation costs of individual electric and plug-in hybrid cars scatter over a wide range in all four scenarios. The small vehicle samples between 2010 and 2014 render it difficult to identify a robust cost trend. Mitigation costs can be particularly high when the CO_2_ emission savings of electric and plug-in hybrid cars relative to their conventional counterparts are small (see calculation method in Equation (7)).
• The median CO_2_ mitigation costs of electric cars tend to decline between 2010 and 2016 in all scenarios.
• Overall, the CO_2_ mitigation costs of electric cars decrease when considering the actual on-road CO_2_ emissions of conventional cars instead of the certified tailpipe emissions; however, the level of CO_2_ mitigation costs of electric cars increase by a factor of 1.3–2.6 when considering well-to-wheel emissions instead of on-road emissions at the tailpipe; the median CO_2_ mitigation costs of electric cars increase by 20–34% when adding the indirect CO_2_ emissions from battery production to the well-to-wheel emissions.
• The median costs for mitigating the certified tailpipe CO_2_ emissions of plug-in hybrids tend to decrease (Fig. 4a), whereas the median costs for mitigating the actual on-road tailpipe emissions show no uniform trend.
• Considering the entire well-to-wheel energy chain, plug-in hybrids tend to emit more CO_2_ than their conventional counterparts (depicted as negative costs in Fig. 4c and d).

By decreasing the carbon intensity of the electricity mix from 707 g CO_2_-equivalents/kWh to 131 g CO_2_-equivalents/kWh through a shift to a renewable electricity supply, the well-to-wheel CO_2_ mitigation costs of electric cars can be decreased by 60%. Likewise, assuming a vehicle lifetime of 11 years (150,000 km) instead of 6 years cuts the CO_2_ mitigation costs by a similar margin. For example, the costs of mitigating real-world CO_2_ tailpipe emissions by electric vehicles decrease from 703 ± 219 EUR_2015_/t CO_2_ to 292 ± 203 EUR_2015_/t CO_2_ under the assumption of an 11 years vehicle lifetime (Table S8 in the Supplementary Material).

#### 3.3.2 Costs of mitigating nitrogen oxides and particle number emissions

**Fig. 5:**
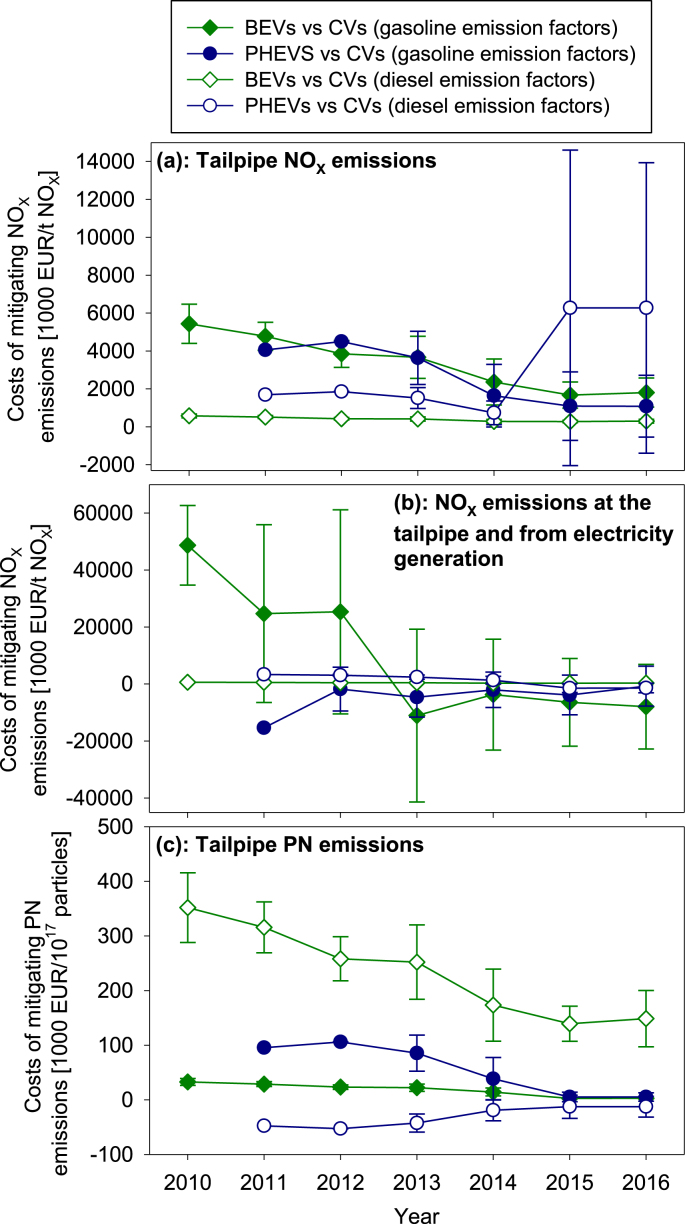
Median costs for mitigating NO_X_ and particle number (PN) emissions of conventional gasoline and diesel cars by electric cars (BEVs) and plug-in hybrids (PHEVs) considering tailpipe emissions (a, c) and a combination of tailpipe emissions and indirect NO_X_ emissions from electricity generation (b); error intervals represent half of the interquartile range of cost data; sample size of one model does not permit presenting an error interval for plug-in hybrid cars in 2011.

Electric and plug-in hybrid cars can mitigate NO_X_ and particle number emissions. The mitigation costs of electric cars tend to decrease from 2010 to 2016 in all three scenarios (Fig. 5); by contrast, the mitigation costs of plug-in hybrids do not show a uniform trend. The mitigation costs incurred by electric cars are particularly low if the comparable conventional cars show high emission levels, as it is the case for NO_X_ emitted by diesel cars (Fig. 5a). The median costs incurred by electric cars decrease by 67% (to 1.8 × 10^6^ EUR/t NO_X_) and 48% (to 3.0 × 10^5^ EUR/t NO_X_) between 2010 and 2016 for mitigating the tailpipe NO_X_ emissions of gasoline and diesel vehicles, respectively. The costs roughly halve to 6.8 × 10^5^ EUR/t NO_X_ and 1.6 × 10^5^ EUR/t NO_X_ when assuming an extended vehicle lifetime of 11 years (Table S8 in the Supplementary Material).

Including the indirect NO_X_ emissions from electricity generation, electric cars (in 2014 and 2016) and plug-in hybrids (in general) emit on average more NO_X_ than their conventional counterparts (see differences between Fig. 5a and b). Following the assumptions in Table 2, plug-in hybrid gasoline cars can mitigate NO_X_ emissions of conventional cars whereas plug-in hybrid diesel cars cannot. If electricity generation is taken into consideration, diesel plug-in hybrids do not save NO_X_ compared to conventional diesel cars (under the assumptions of Table 2).

The costs for mitigating particle number emissions by electric cars decreased between 2010 and 2016 on average by 92% (from 3.3 × 10^4^ EUR/10^17^ particles to 2.7 × 10^3^ EUR/10^17^ particles) when considering gasoline cars and 58% (from 3.5 × 10^5^ EUR/10^17^ particles to 1.5 × 10^5^ EUR/10^17^ particles) when considering diesel cars, respectively. The higher costs of electric cars to mitigate particle emissions of diesel cars compared to those of gasoline cars stem from the high emissions factor for gasoline cars without particulate filters (see Table 2). Plug-in hybrids can hardly mitigate particle emissions and may even show higher emission levels than conventional cars (see emission factors in Table 2).

## 4 Discussion

### 4.1 Discussion of price trends and experience curves

#### 4.1.1 Limitations and uncertainty

The present analysis comprises all models of mass-produced electric and plug-in hybrid cars sold in Germany between 2010 and 2016. As the German car market is competitive, similar price trends are likely to be found also on other competitive vehicle markets such as China, Japan, and the USA. The learning rates on these markets can, however, differ somewhat from those identified here as manufacturers may alter the market positioning of models to match local purchasing power and consumers taste.

Our analysis does not distinguish between *parallel* and *series* plug-in hybrids. This choice may introduce uncertainty analysis because the relative frequency of comparatively expensive *parallel* plug-in hybrids varies in the data samples for individual years (see Table S4 in the Supplementary Material).

Moreover, electric cars were excluded if their traction battery is offered through a lease contract. Battery leasing lowers the initial price of electric cars and absorbs consumer uncertainty about battery durability, which, in turn, can decrease the implicit consumer discount rate for electric cars (Sigrin, 2013; Liao et al., 2017; Haq and Weiss, 2018). While sold and leased batteries are subject to similar rates of technological learning, we see merits in surveys eliciting consumer preferences for purchasing versus leasing traction batteries to obtain insight into persisting market barriers for electric cars.

Our experience curve analysis is subject to caveats related, e.g., to the approximation of production costs by market prices or inhomogeneity of vehicle attributes that are discussed in Text Box 2 in the Supplementary Material.

#### 4.1.2 Implications for science and policy

The learning rates identified here for the price (23 ± 2%) and price differential of electric cars (32 ± 2%) exceed: (i) the those identified by Safari (2017) for the price of electric cars (9%) and the costs of powertrain electrification excluding battery (12%) as well as (ii) the 8 ± 1% identified by Weiss et al. (2015) for the price of e-bikes. However, the learning rates for the price (6 ± 1%) and price differential (37 ± 2%) of plug-in hybrids confirm, in part, the learning rates of 7 ± 2% and 23 ± 5% (mean ± 95% confidence interval) identified for non-plug-in hybrid cars by Weiss et al., 2012a, Weiss et al., 2012b.

The high learning rates for electric cars suggest rapid technological learning in the manufacturing of traction batteries, which constitute the largest individual cost component of an electric powertrain (Safari, 2017). Together with other electric powertrain components, the traction battery constitutes a higher share in the overall production costs of electric cars than it does in the production costs of plug-in hybrids. Nagelhout and Ros (2009) as well as Nykvist and Nilsson (2015) identified learning rates of 17% and 6–9%, respectively for the manufacturing of lithium-ion batteries. IRENA (2017a) expects the costs for these batteries will decrease to below 100 USD/kWh within a decade. AMS (2017) sketches an even more optimistic scenario, stipulating that industry to date already operates with costs of 100 EUR/kWh battery capacity. As technological learning in battery manufacturing is not limited to the automotive industry, spill-overs of economy-wide battery applications, including stationary applications in buildings, may increasingly benefit vehicle batteries in the future. Volatility in lithium prices may not significantly affect these costs in the midterm (Ciez and Whitacre, 2016) as raw materials (lithium and others) account for only 12% of the manufacturing costs of lithium-ion batteries (Helmers, 2015).

Safari (2017) found that 37 ± 2% of the electrification costs and 19 ± 1% of the total manufacturing costs of electric cars stem from the traction battery. Thus, modules such as the electric motor, power electronics, and auxiliary components (Safari, 2017) together offer a large potential for technological learning independent from battery manufacturing.

If technological learning continues to decrease production costs, vehicle prices will soon become a minor barrier for the market penetration of electric cars. Moreover, high prices do not *per se* prohibit the market penetration of status revealing commodities such as passenger cars. We think the deployment of electric and plug-in hybrid cars could benefit greatly from branding, marketing, and clever product positioning that exploits status competition and social frames of consumers (Haq and Weiss, 2018). Such strategies can, however, be effective only if non-cost barriers including short drive ranges, long recharging times, and inconvenient recharging infrastructure are addressed. The recent experience in Germany seems to support this argument: In the 11 months since subsidies of 4000 EUR and 3000 EUR are granted per electric and plug-in hybrid car (BR, 2016b), just 20,000 applications for receiving a subsidy were submitted (AB, 2017a). This low number is remarkable because the level of the subsidy overcompensates, on average, the price difference between electric cars (214 ± 237 EUR/kW) and plug-in hybrids (20 ± 38 EUR/kW) and their conventional counterparts. Consistently, Lévay et al. (2017) did not identify a clear link between the level of subsidies and the number of electric cars sold in several European countries. It is therefore reasonable to expect that part of the subsidy is ineffective and invites wasteful free-riding (see also Hardman et al., 2017). To ensure effective policy support for electric vehicles, regulators and industry could:

• reconsider subsidies and focus on non-cost market barriers;
• address the still limited consumer experience with electric cars and decrease risk aversion and transaction costs by offering attractive leasing schemes, extended warranty, maintenance, take-back plans, or recharging facilities at work places, public parking areas, or car dealerships, whose reluctance to promote electric and plug-in hybrid cars appears to be an important obstacle for the electrification of road transport in Germany (AB, 2017b);
• tighten CO_2_ emissions targets for passenger cars, such as the 95 g/km fleet-average target in the EU (EC, 2009).

### 4.2 Discussion of user costs

#### 4.2.1 Limitations and uncertainty

The user costs reflect the set of specific assumptions made here. The assumption of a 6-year vehicle lifetime equates to an average mileage of 86,000 km, which is less than the 170,000–230,000 km lifetime mileage observed for passenger cars in Germany (Weymar and Finkbeiner, 2016). As our analysis may thus over-emphasize the contribution of the vehicle price to the overall user costs, we also consider in a sensitivity analysis an extended lifetime of 11 years (150,000 km). This analysis reflects the use pattern of vehicles in Germany (Weymar and Finkbeiner, 2016) but it excludes the cost of battery replacement and could therefore underestimate the user costs of electric and plug-in hybrid cars.

For plug-in hybrids, a deviation between certified and real-world fuel consumption of 218% was assumed based on a sample of 1135 vehicles presented by Tietge et al. (2016). The assumed deviation seeks to capture the average use condition of these vehicles that is subject to considerable variability as recharging patterns can vary from frequent to never. In cases where plug-in hybrids are frequently recharged, and thus driven largely electrically, the assumption of a 218% divergence overestimates fuel consumption and the costs of mitigating emissions (see also Section 3.1).

#### 4.2.2 Implications for science and policy

User costs scatter over a wide range (Fig. 3a) but do not decline in the same way as the price and price differentials of electric cars and plug-in hybrids do (Fig. 2). This observation suggests manufacturers deploy increasingly larger, more expensive, and less efficient cars. Electric cars and plug-in hybrids thereby follow the general market trend (ICCT, 2017; Weiss et al., 2019), which in turn, supports our previous argument that current price and cost levels may constitute a minor barrier for the market penetration of electric vehicles.

### 4.3 Discussion of emissions mitigation costs

#### 4.3.1 Limitations and uncertainty

Also the costs for mitigating emissions reflect the assumptions made here. The cost estimates scatter over a wide range and can assume very high absolute values if emission savings of electric and plug-in hybrid cars are close to zero (see Equation (7)). Moreover, mitigation costs become negative if either savings in user costs or savings in emissions are negative, which renders cost estimates ambiguous. If both savings in user costs and emissions are negative, the result becomes positive and depicts the costs accrued by conventional cars for mitigating the emissions from electric and plug-in hybrid cars. Given these intricacies, it is important to inspect the emissions mitigation costs and their underlying data carefully before drawing conclusions. Our calculation method yields robust results in cases where an expensive novel technology yields substantial emission savings (as is the case for electric cars mitigating the tailpipe NO_X_ emissions of diesel cars; see Fig. 5a). However, if costs and emissions of a novel technology are similar to those of the incumbent technology (as is the case for plug-in hybrid diesel cars replacing conventional diesel cars in Fig. 4, Fig. 5), the results may not be robust.

The extent, to which electric cars and plug-in hybrids can mitigate the emissions of conventional cars, depends on the assumed emission factors. Certified CO_2_ emissions at the tailpipe are determined in a standardized procedure; the respective emission factors are therefore robust. However, the CO_2_ emissions on the road depend on the actual vehicle operation and can scatter over a wider range. This paper does not account for this variability but captures the average deviation between certified and on-road CO_2_ emissions (Tietge et al., 2016). This average cannot obviously reflect the specific CO_2_ emissions of each car model under any conceivable operating conditions. The resulting uncertainty is specifically high for plug-in hybrids whose tailpipe CO_2_ emissions can vary between zero to above the levels of conventional cars depending on the charging status of the traction battery.

The assumed carbon intensity of the electricity mix (707 g CO_2_-equivalents/kWh) captures the average situation in Germany as of 2013 and includes own consumption of power plants and well-to-plug losses in the electricity system (Helmers et al., 2017). The assumed value is therefore higher than the carbon intensity of 573 g CO_2_/kWh as reported by UBA (2018) for the same year.

Given the limited data availability, the assumed NO_X_ and particle number emission factors (Table 2) require scrutiny from further emissions testing (see also Text Box 4 in the Supplementary Material).

Finally, our cost analysis provides an indication of the average marginal costs incurred by electric cars and plug-in hybrids sold in Germany for mitigating CO_2_ and air pollutant emissions below the emission levels of conventional cars. Given the large variability in user costs and actual on-road emissions, the average emission mitigation costs may thus not represent adequately the cost performance of each individual vehicle.

#### 4.3.2 Implications for science and policy

Electric and plug-in hybrid cars operated in Germany can mitigate tailpipe CO_2_ emissions at median costs of 700 ± 200 EUR/t CO_2_ (electric cars) and 1400 ± 1600 EUR/t CO_2_ (plug-in hybrids). The median costs for electric cars level at 1700 ± 1000 EUR/t CO_2_ if indirect emissions of electricity generation are accounted for; these costs could decrease to 680 ± 220 EUR/t CO_2_ if electricity was generated by renewables. The cost levels in all scenarios could decrease by more than 50% when assuming a vehicle lifetime of eleven years instead of six years (see Table S8 in the Supplementary Material). These values are broadly in line with the costs of 2000–2500 EUR/t CO_2_-equivalents found by ASUE (2016) for electric cars driven 15,000 km per year. The Emissions mitigation costs are higher than/in line with the 400–600 EUR/t CO_2_-equivalents determined by ASUE (2016) when assuming a 6/11 year lifetime of vehicles. Depending on the scenario considered, CO_2_ emissions mitigation costs of electric cars and plug-in hybrids already to date approach cost levels of <100 EUR/t CO_2_ as projected by McKinsey (2009) for the year 2030.

The CO_2_ emissions mitigation costs of electric and plug-in hybrid cars are: (i) high when assuming a 6-year vehicle lifetime and (ii) comparable when assuming an 11-year lifetime with renewable energies like wind and photovoltaics that can to date already save CO_2_ emissions at no additional costs compared to fossil energy resources (Boshell et al., 2017; IRENA, 2018).

**Fig. A1:**
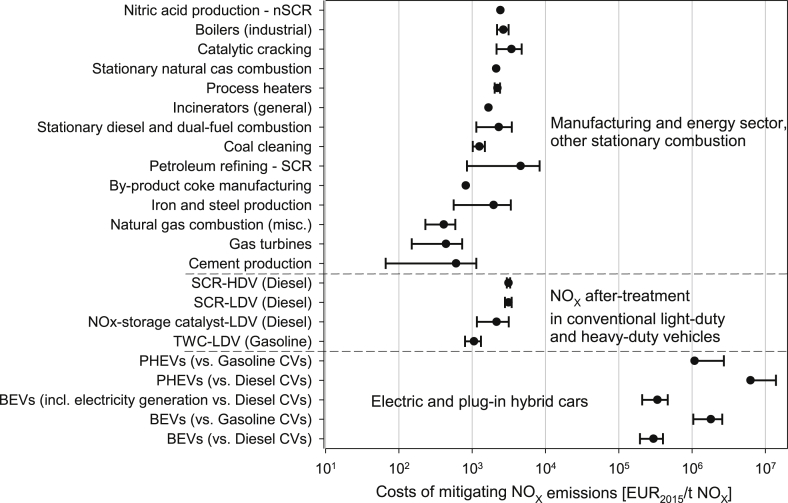
Indicative costs of mitigating NO_X_ emissions assuming a 6 years vehicle lifetime; dots and error intervals depict: (i) the midpoint and range of costs of after-treatment technologies in cars and stationary installations and (ii) the median and half of the interquartile range of costs for electric and plug-in hybrid cars in 2016; negative costs for electric cars and plug-in hybrids are not shown; SCR – selective catalytic reduction; nSCR - non-selective catalytic reduction; LDV – light-duty vehicle; HDV – heavy-duty vehicles; TWC – three-way catalyst; data sources: EPA (2015) and Zerfass (2017). The NO_X_ mitigating potential of plug-in hybrids compared to conventional cars is negligible given the assumptions in Table 2. The same applies to electric cars compared to gasoline vehicles if the NO_X_ emissions from electricity generation are included. The costs for mitigating NO_X_ emissions in these two cases can assume very large negative or positive values following our calculation method in Equation (7) (see also Section 4.4.1).

**Fig. A2:**
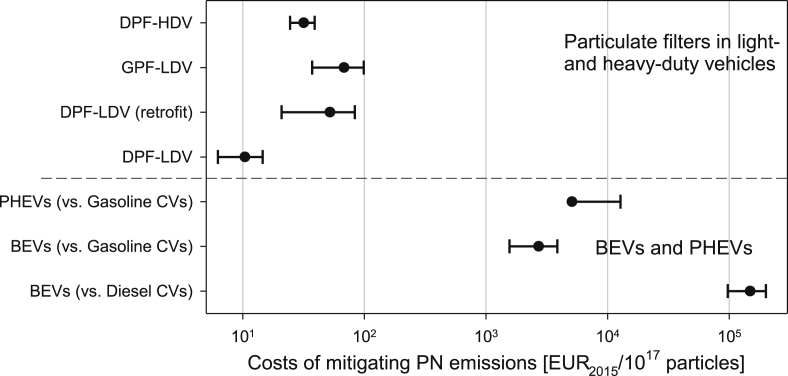
Indicative costs of mitigating particle number (PN) tailpipe emissions of conventional cars (CV) through the deployment of electric cars (BEVs) and plug-in hybrid cars (PHEVs) assuming a 6 years vehicle lifetime; dots and error intervals depict: (i) the midpoint and range of costs for particulate filters and (ii) the median and half of the interquartile range of cost for electric and plug-in hybrid cars for the year 2016; negative costs for electric cars and plug-in hybrids are not shown; DPF – diesel particulate filter, GPF – gasoline particulate filter; LDV – light-duty vehicle; HDV – heavy-duty vehicles; CV - conventional car; data source: Zerfass (2017). Diesel plug-in hybrids do not mitigate PN emissions relative to conventional diesel cars following the assumptions in Table 2 in the main text.

Whereas the costs for mitigating CO_2_ emissions are comparable to other technologies, the costs for mitigating NO_X_ and particle emissions by electric cars and plug-in hybrids are several orders of magnitudes higher than those incurred by: (i) after-treatment technologies of conventional cars (800–3800 EUR/t NO_X_; 6–100 EUR/10^17^ particles; Figs. A1 and A2; Table S8 in the Supplementary Material). Care is however necessary when interpreting this observation as cost levels are subject to a proper functioning of after-treatment systems (see Text Box 3 in the Supplementary Material). The NOx and PN emissions mitigation costs of electric and plug-in hybrid cars decrease but are still high compared to after-treatment technologies when an extended lifetime of eleven years is assumed. This observation suggests economic merits in advancing emissions control technologies of conventional cars such as selective-catalytic reduction (SCR) that is readily available^1^ and whose application would allow meeting the applicable air quality standards in Europe (Degraeuwe et al., 2017). To realize the existing potentials of after-treatment technologies necessitates a rigorous enforcement of existing emission legislation. If done, the current levels of urban NO_2_ and particle pollution are decreased less costly through catalysts and filters than through the deployment of electric and plug-in hybrid cars.

## 5 Conclusions

We draw the following conclusions:

• Electric and plug-in hybrid cars have become cheaper and more cost competitive since their introduction into the mass-vehicle market in 2010.
• The robust price decline suggests substantial technological learning that will likely continue to decrease the production costs and prices of electric and plug-in hybrid cars in the future.
• Electric cars show higher learning rates than plug-in hybrids, which indicates considerable technological learning in the manufacturing of batteries and other electric powertrain components.
• The user costs of electric and plug-in hybrid cars scatter over a wide range; the costs tend to increase on average, following a trend towards larger, more powerful, and thus less efficient, cars. However, the mean cost differentials between electric and plug-in hybrid cars and their conventional counterparts are declining.
• The substantial decline in the price of electric cars and plug-in hybrids in conjunction with a market trend towards larger and more powerful vehicles suggest high prices and costs may no longer be the primary factor inhibiting the electrification of road transport. If so, policy makers and industry could reconsider subsidies and focus on non-cost market barriers such as: (i) drive range, recharging times, and availability of recharging infrastructure, (ii) warranty, maintenance, and take-back plans, (iii) branding, marketing, and product positioning that capitalizes on the status competition and social frames of consumers.
• The costs for mitigating CO_2_ and air pollutant emissions by electric and plug-in hybrid cars scatter over wide ranges and are specific to the set of assumptions applied here.
• Electric cars can mitigate CO_2_ and pollutant emissions, even when considering the indirect emissions from electricity generation and battery production. The CO_2_ mitigation costs will likely continue to decrease in the future through technological learning and a growing share of renewables in the electricity mix.
• The costs for mitigating NO_X_ and particle emissions by electric and plug-in hybrid cars decline but are comparatively high. At current levels, NO_X_ and particle emissions are mitigated less costly through state-of-the-art after-treatment systems than through the electrification of powertrains.

## Declarations of interest

None.

## Footnotes

1 Analyses conducted in the aftermath of the Diesel-NO_X_ scandal suggest a widespread application of defeat devises that deactivate or decrease the effectiveness of after-treatment systems during normal vehicle use (BMVI, 2016; Degraeuwe and Weiss, 2017). The costs of mitigating NO_X_ emissions through inactive emission control technologies are infinite.
