# Supplementary material for: Fully electric and plug-in hybrid cars - An analysis of learning rates, user costs, and costs for mitigating CO2 and air pollutant emissions
Source: J Clean Prod. 2019 Mar 1;212:1478–89. doi: 10.1016/j.jclepro.2018.12.019 (PMC6358050; doi:10.1016/j.jclepro.2018.12.019)
Supplement: Supplementary_Material_Tables [file mmc1.docx]

Table S5: Explanations on the data used for calculating user costs and the costs for mitigating CO_2_ and NO_X_ emissions through electric and plug-in hybrid cars in Germany (numbers in the table are identical with those in Table 1)

| Parameter (Source) | Electric cars | Plug-in hybrid cars | Conventional  cars |
| --- | --- | --- | --- |
| Lifetime [a] (BMF, 2017); assumption for sensitivity analysis in parentheses | 6 (11)^h^ | 6 (11)^h^ | 6 (11)^h^ |
| Yearly mileage [km] (KBA, 2015) | 14,259 | 14,259 | 14,259 |
| Electricity price [EUR/kWh] (BDEW, 2017) | 0.27^a^ | 0.27^a^ | - |
| Fuel price [EUR_2015_/l] (Statista, 2017b,c) | - | 1.31 (diesel)^f^  1.49 (gasoline)^f^ | 1.31 (diesel)^f^  1.49 (gasoline)^f^ |
| Carbon intensity of the electricity mix  [g CO_2_-equivalents/kWh] (Helmers et al., 2017) | 707^b^ | - | - |
| Well-to-tank fuel losses [% of CO_2_ emissions at the tailpipe] (Fritsche, 2007) | - | 18 | 18 |
| Correction factor for the difference between certified and real-world electricity and fuel consumption [% of certified value] | 30^c^ | 218^d^ | year-specific estimates^e^ |
| NO_X_ emissions of power generation [g/kwh] (Helmers, 2010)^g^ | 0.44 | 0.44 | - |
| Carbon emissions of battery production [kg CO_2_-equivalents/kWh] (Moro and Helmers, 2017) | 168 | 168 | - |

^a^mean electricity price as given by BDEW (2017) for the period between 2010 and 2016

^b^considering the average electricity mix in Germany, including transmission losses from plant to plug and own consumption of power plants

^c^based on Zerfass (2015)

^d^Estimate represents the weighted average deviation between certified and real-world fuel consumption as calculated by the authors based on Tietge et al. (2016) who capture 1135 plug-in hybrids of which: 133 vehicles were driven in the UK, 995 vehicles were driven in the Netherlands, and 7 vehicles were driven in Germany. Although plug-in hybrids exhibit on average a high deviation between certified and real-world fuel consumption and CO_2_ emissions, charging pattern have a tremendous impact on the actual divergence experienced by individual vehicles users. As frequent recharging can decrease the tail-pipe emissions of plug-in hybrids to zero, the estimate used for our cost analysis may represent the average use pattern but not the specific use pattern of each individual plug-in hybrid car.

^e^The deviation between certified and actual fuel consumption of conventional cars is not constant but tends to increase over time. The year-specific estimates used here are obtained from Figure ES-1 in Tietge et al. (2016).

^f^mean diesel and gasoline price as given by Statista (2017a,b) for the period between 2010 and 2016

^g^the NO_X_ intensity of the German electricity mix has remained relatively constant in the period between 2000 and 2015 despite the deployment of renewables (UBA, 2017)

^h^In a sensitivity analysis (see Table S5 in the Supplementary Material), we assume an extended lifetime of 11 years (150,000 km) that is consistent with the lifetime mileage driven by cars in Germany (Weymar and Finkbeiner, 2016).

Table S6: Generic NO_X_ and particle number tailpipe emission factors of electric, plug-in hybrid, and conventional cars; principal data sources: EEA (2016b), Giechaskiel et al. (2015) and Hammer et al. (2015) for NO_X_ and particle number emission factors, respectively (numbers in the table are identical with those in Table 2)

| Pollutant | NO_X_ [mg/km] | Particle number [#/km] |
| --- | --- | --- |
| Electric cars | - | - |
| Plug-in hybrid cars - Gasoline (Euro 5) | 13 | 8×10^11a^ |
| Plug-in hybrid cars - Gasoline (Euro 6b) | 13 | 3×10^12b^ |
| Plug-in hybrid cars - Diesel (Euro 5) | 490^f^ | 8×10^11g^ |
| Plug-in hybrid cars - Diesel (Euro 6b) | 490^f^ | 8×10^11g^ |
| Conventional cars - Diesel (Euro 5) | 610 | 4×10^11c^ |
| Conventional cars - Diesel (Euro 6b) | 500 | 4×10^11c^ |
| Conventional cars - Gasoline (Euro 5) | 60 | 1×10^12d^ |
| Conventional cars - Gasoline (Euro 6b) | 60 | 4×10^12e^ |

^a^based on the particle number emissions of one car equipped with a port-fuel-injection engine and the assumption that plug-in hybrids drive 20% of the distance electrically

^b^based on the midpoint of particle number emissions observed for seven vehicles with gasoline direct injection engines and the assumption that plug-in hybrids drive 20% of the distance electrically

^c^based on the mean particle number emissions of two diesel cars equipped with a particulate filter

^d^based on the particle number emissions of one car equipped with a port-fuel-injection engine

^e^based on the midpoint of particle number emissions observed for seven vehicles with gasoline direct injection engines

^f^based on two plug-in hybrid diesel cars tested by Franco et al. (2016)

^g^conservative estimate based on expert judgment and two plug-in hybrid diesel cars tested by Hammer et al. (2015)

Table S7: Mean user costs of electric, plug-in hybrid, and conventional cars when assuming a vehicle lifetime of 6 *versus* 11 years; error intervals depict the standard deviation of cost data

|  | Year | 2010 | 2011 | 2012 | 2013 | 2014 | 2015 | 2016 |
| --- | --- | --- | --- | --- | --- | --- | --- | --- |
| User costs [EUR_2015_/km] | Electric cars - 6 years lifetime | 0.64 ± 0.18 | 0.56 ± 0.10 | 0.55 ± 0.09 | 0.65 ± 0.28 | 0.57 ± 0.17 | 0.54 ± 0.18 | 0.74 ± 0.46 |
|  | Electric cars - 11 years lifetime | 0.43 ± 0.10 | 0.39 ± 0.06 | 0.38 ± 0.05 | 0.46 ± 0.19 | 0.41 ± 0.12 | 0.38 ± 0.12 | 0.51 ± 0.30 |
|  | Plug-in hybrid cars - 6 years lifetime | - | 0.76^a^ | 0.71 ± 0.10 | 0.95 ± 0.56 | 1.12 ± 0.53 | 1.09 ± 0.42 | 1.06 ± 0.41 |
|  | Plug-in hybrid cars - 11 years lifetime | - | 0.53^a^ | 0.50 ± 0.06 | 0.67 ± 0.38 | 0.78 ± 0.34 | 0.77 ± 0.28 | 0.75 ± 0.27 |
|  | Conventional cars, comparable to electric cars - 6 years lifetime | 0.31 ± 0.04 | 0.31 ± 0.04 | 0.33 ± 0.06 | 0.45 ± 0.22 | 0.45 ± 0.18 | 0.44 ± 0.17 | 0.61 ± 0.40 |
|  | Conventional cars, comparable to electric cars - 11 years lifetime | 0.26 ± 0.03 | 0.25 ± 0.03 | 0.27 ± 0.04 | 0.36 ± 0.15 | 0.35 ± 0.13 | 0.35 ± 0.12 | 0.47 ± 0.28 |
|  | Conventional cars, comparable to plug-in hybrids - 6 years lifetime | - | 0.55^a^ | 0.49 ± 0.09 | 0.79 ± 0.48 | 1.01 ± 0.59 | 1.01 ± 0.47 | 1.01 ± 0.47 |
|  | Conventional cars, comparable to plug-in hybrids - 11 years lifetime | - | 0.43^a^ | 0.38 ± 0.07 | 0.58 ± 0.31 | 0.73 ± 0.39 | 0.73 ± 0.32 | 0.73 ± 0.30 |

- no data available

^a^only one data point available

Table S8: Costs of electric cars and plug-in hybrids to mitigate the CO_2_ and air pollutant emissions of conventional cars; values and error margins depict the median and half of the interquartile range of cost data; the interpretation of negative values is not straight forward and requires careful inspection of the underlying user costs and emission factors

| Year | 2010 | 2011 | | 2012 | | 2013 | 2014 | 2015 | 2016 |
| --- | --- | --- | --- | --- | --- | --- | --- | --- | --- |
| Emissions mitigation costs in [EUR_2015_/t CO_2_] | CO_2_ emissions | | | | | | | | |
|  | Scenario I: Certified tail-pipe emissions | | | | | | | | |
| Electric cars - 6 years lifetime | 3026 ± 433 | 2538 ± 288 | | 2331 ± 443 | | 1876 ± 440 | 1342 ± 601 | 1008 ± 512 | 999 ± 312 |
| Electric cars - 11 years lifetime | 1660 ± 256 | 1378 ± 249 | | 1188 ± 240 | | 1017 ± 287 | 682 ± 407 | 376 ± 379 | 414 ± 289 |
| Plug-in hybrid cars - 6 years lifetime | - | 1443^a^ | | 3425 ± 903 | | 1376 ± 1015 | 750 ± 706 | 527 ± 750 | 505 ± 668 |
| Plug-in hybrid cars - 11 years lifetime | - | 628^a^ | | 1738 ± 505 | | 655 ± 630 | 282 ± 459 | 152 ± 485 | 137 ± 418 |
|  | Scenario II: Real-world tailpipe emissions on the road | | | | | | | | |
| Electric cars - 6 years lifetime | 2402 ± 330 | 1976 ± 224 | | 1786 ± 339 | | 1380 ± 324 | 959 ± 430 | 710 ± 361 | 703 ± 219 |
| Electric cars - 11 years lifetime | 1308 ± 188 | 1073 ± 194 | | 911 ± 184 | | 748 ± 211 | 487 ± 291 | 265 ± 267 | 292 ± 203 |
| Plug-in hybrid cars - 6 years lifetime | - | 1786^a^ | | 32 ± 966 | | 543 ± 1793 | 915 ± 1105 | 2179 ± 2940 | 1398 ± 1574 |
| Plug-in hybrid cars - 11 years lifetime | - | 878^a^ | | -39 ± 505 | | 105 ± 1115 | 459 ± 730 | 1233 ± 1845 | 808 ± 1179 |
|  | Scenario III: Well-to-wheel emissions | | | | | | | | |
| Electric cars - 6 years lifetime | 8762 ± 3090 | 4838 ± 2211 | | 6406 ± 3455 | | 4017 ± 1320 | 2198 ± 2239 | 1865 ± 1148 | 1710 ± 995 |
| Electric cars - 11 years lifetime | 4666 ± 1842 | 2251 ± 1401 | | 3493 ± 1964 | | 1972 ± 700 | 986 ± 1434 | 599 ± 668 | 601 ± 694 |
| Plug-in hybrid cars - 6 years lifetime | - | 13533^a^ | | 6895 ± 3992 | | -662 ± 1788 | -1017 ± 827 | -1190 ± 709 | -744 ± 539 |
| Plug-in hybrid cars - 11 years lifetime | - | 6651^a^ | | 3379 ± 1991 | | -713 ± 775 | -937 ± 447 | -915 ± 390 | -672 ± 371 |
|  | Scenario IV: Emissions along the well-to-wheel chain and from battery manufacturing | | | | | | | | |
| Electric cars - 6 years lifetime | -1727 ± 92134 | 5912 ± 13193 | | 5728 ± 17934 | | 4464 ± 12278 | 3304 ± 9579 | 1635 ± 5850 | -1284 ± 5611 |
| Electric cars - 11 years lifetime | 5271 ± 5232 | 2265 ± 3125 | | 5954 ± 2029 | | 2609 ± 1972 | 790 ± 1773 | 706 ± 1323 | 1200 ± 1651 |
| Plug-in hybrid cars - 6 years lifetime | - | -13392^a^ | | -7884 ± 3420 | | -1947 ± 1317 | -1478 ± 784 | -1027 ± 521 | -932 ± 525 |
| Plug-in hybrid cars - 11 years lifetime | - | -45333^a^ | | -25379 ± 12396 | | -1626 ± 3123 | -1047 ± 377 | -730 ± 381 | -609 ± 475 |
| Emissions mitigation costs in [1000 EUR_2015_/  t NO_X_] | NO_X_ emissions | | | | | | | | |
|  | Scenario I: Real-world tailpipe emissions on the road | | | | | | | | |
| Electric cars versus conventional gasoline cars -  6 years lifetime | 5436 ± 1035 | | 4779 ± 740 | | 3846 ± 709 | 3664 ± 1112 | 2360 ± 1216 | 1670 ± 690 | 1807 ± 766 |
| Electric cars versus conventional gasoline cars -  11 years lifetime | 2955 ± 515 | | 2181 ± 378 | | 2075 ± 406 | 1911 ± 520 | 1080 ± 845 | 679 ± 588 | 680 ± 497 |
| Electric cars versus conventional diesel cars -  6 years lifetime | 576 ± 105 | | 517 ± 76 | | 423 ± 66 | 413 ± 112 | 284 ± 108 | 279 ± 64 | 297 ± 103 |
| Electric cars versus conventional diesel cars -  11 years lifetime | 332 ± 54 | | 262 ± 41 | | 249 ± 40 | 241 ± 53 | 158 ± 76 | 150 ± 55 | 158 ± 50 |
| Plug-in hybrid cars versus conventional gasoline cars - 6 years lifetime | - | | 4052^a^ | | 4499 ± 23 | 3635 ± 1405 | 1637 ± 1652 | 1087 ± 1809 | 1083 ± 1629 |
| Plug-in hybrid cars versus conventional gasoline cars - 11 years lifetime | - | | 1764^a^ | | 2192 ± 74 | 1699 ± 999 | 635 ± 1071 | 317 ± 1134 | 309 ± 1052 |
| Plug-in hybrid cars versus conventional diesel cars - 6 years lifetime | - | | 1689^a^ | | 1855 ± 14 | 1517 ± 548 | 735 ± 625 | 6274 ± 8325 | 6273 ± 7667 |
| Plug-in hybrid cars versus conventional diesel cars - 11 years lifetime | - | | 793^a^ | | 951 ± 24 | 759 ± 388 | 343 ± 397 | 2649 ± 5294 | 2637 ± 5033 |
|  | Scenario II: Real-world tailpipe emissions on the road and emissions from electricity generation | | | | | | | | |
| Electric cars versus conventional gasoline cars -  6 years lifetime | 48687 ± 13981 | | 24721 ± 31174 | | 25335 ± 35810 | -11065 ± 30292 | -3692 ± 19468 | -6416 ± 15380 | -7934 ± 14843 |
| Electric cars versus conventional gasoline cars -  11 years lifetime | 23813 ± 6823 | | 11282 ± 14980 | | 11576 ± 17750 | -4035 ± 15161 | 1320 ± 9180 | -562 ± 7146 | -1471 ± 5880 |
| Electric cars versus conventional diesel cars -  6 years lifetime | 647 ± 124 | | 56 2±73 | | 467 ± 70 | 463 ± 119 | 312 ± 123 | 321 ± 74 | 336 ± 128 |
| Electric cars versus conventional diesel cars -  11 years lifetime | 373 ± 64 | | 284 ± 38 | | 273 ± 42 | 270 ± 57 | 175 ± 88 | 169 ± 60 | 182 ± 61 |
| Plug-in hybrid cars versus conventional gasoline cars - 6 years lifetime | - | | -15358^a^ | | -1782 ± 7722 | -4644 ± 6938 | -2047 ± 6203 | -3842 ± 6961 | -736 ± 7002 |
| Plug-in hybrid cars versus conventional gasoline cars - 11 years lifetime | - | | -6685^a^ | | -288 ± 3731 | -284 ± 4100 | -30 ± 4011 | -1334 ± 4020 | 1166 ± 3491 |
| Plug-in hybrid cars versus conventional diesel cars - 6 years lifetime | - | | 3345^a^ | | 3105 ± 311 | 2413 ± 770 | 1341 ± 1332 | -1430 ± 1561 | -1413 ± 1703 |
| Plug-in hybrid cars versus conventional diesel cars - 11 years lifetime | - | | 1571^a^ | | 1574 ± 108 | 1179 ± 599 | 621 ± 832 | -573 ± 1013 | -564 ± 1044 |
| Emissions mitigation costs in [1000 EUR_2015_/10^17^ particles] | PN emissions | | | | | | | | |
|  | Scenario: Real-world tail-pipe emissions on the road | | | | | | | | |
| Electric cars versus conventional gasoline cars -  6 years lifetime | 33 ± 6 | 29 ±4 | | 23 ± 4 | | 22 ± 7 | 14 ± 7 | 3 ± 1 | 3 ± 1 |
| Electric cars versus conventional gasoline cars -  11 years lifetime | 18 ± 3 | 13 ± 2 | | 12 ± 2 | | 11 ± 3 | 6.5 ± 5.1 | 1.0 ± 0.9 | 1.0 ± 0.7 |
| Electric cars versus conventional diesel cars -  6 years lifetime | 352 ± 64 | 316 ± 46 | | 258 ± 40 | | 252 ± 68 | 173 ± 66 | 139 ± 32 | 149 ± 52 |
| Electric cars versus conventional diesel cars -  11 years lifetime | 203 ± 33 | 160 ± 25 | | 152 ± 24 | | 147 ± 33 | 97 ± 47 | 75 ± 27 | 79 ± 25 |
| Plug-in hybrid cars versus conventional gasoline cars - 6 years lifetime | - | 95^a^ | | 106 ± 1 | | 85 ± 33 | 38 ± 39 | 5 ± 9 | 5 ± 8 |
| Plug-in hybrid cars versus conventional gasoline cars - 11 years lifetime | - | 41^a^ | | 52 ± 2 | | 40 ± 23 | 15 ± 25 | 1.5 ± 5.3 | 1.5 ± 4.9 |
| Plug-in hybrid cars versus conventional diesel cars - 6 years lifetime | - | -48^a^ | | -52.9 ± 0.3 | | -43 ± 17 | -19 ± 19 | -13 ± 22 | -13 ± 19 |
| Plug-in hybrid cars versus conventional diesel cars - 11 years lifetime | - | -21^a^ | | -26 ± 1 | | -20 ± 12 | -7 ± 13 | -4 ± 13 | -4 ± 12 |

- no data available

^a^only one data point available

Table S9: Data sources and assumptions used for estimating the costs of mitigating NO_X_ emissions of road vehicles and the manufacturing industry; data represent estimates that indicate general cost trends (Source: Zerfass, 2017)

| **Technology^d^** | **Costs** | **Lifetime** | **Efficacy** | **Mitigation costs** |
| --- | --- | --- | --- | --- |
| Light-duty vehicles -  Three-way catalyst  (including sensors and control software) | 230-380 EUR (estimate based on Possada et al., 2012)^a,b^ | 240,000 km (based on Tier 3 durability requirements in the USA; Delphi, 2015) | 95% NO_X_ removal (based on Mooney (2007) | 840-1,400 EUR/t NOx |
| Light-duty vehicles –  Lean NO_X_-storage catalyst | 250-680 EUR (estimate based on Possada et al., 2012)^a,b^ | 240,000 km based on Tier 3 durability requirements in the US (Delphi, 2015) | NO_X_ reduction from 1,20 g/km to 0.30 g/km = 0,90 g/km (first order estimate of the authors) | 1,200-3,200 EUR/t NO_X_ |
| Light-duty vehicles –  Selective catalytic reduction | System costs: 330-500 EUR estimate based on Possada et al., 2012)^a,b^;  Urea costs: 1.65 EUR/1000 km^c^ | 240,000 km based on Tier 3 durability requirements in the US (Delphi, 2015) | NO_X_ reduction from 1.20 g/km to 0.12 g/km = 1,08 g/km (estimate of the authors) | 2,800-3,800 EUR/t NOx |
| Heavy-duty vehicles –  Selective catalytic reduction | 1,830-2,510 EUR (estimate of manufacturing costs for a vanadium-based SCR system based on Possada, et al., 2016)^a^; urea costs: 0,88 EUR/100 km^c^ | 500,000 km based on Euro VI durability requirements (Delphi, 2017) | NO_X_ reduction to 0.21 g/km (ICCT, 2016); NO_X_ reduction efficiency of 95% (first order estimate of the authors) | 3,100-3,500 EUR/t NO_X_ |
| Cement production –  Various technologies (bio-solid injection, selective non-catalytic reduction, mid-kiln firing) | - | - | - | 66-1,140 EUR/t NO_X_^f^  (EPA; 2015) |
| Gas turbines –  low NO_X_ burner | - | - | - | 150-730 EUR/t NO_X_^f^  (EPA; 2015) |
| Natural gas – pipeline compressors and stationary combustion (miscellaneous technologies including non-selective catalytic reduction, low-emission combustion, ignition retardation, adjusted air-to-fuel ratio) | - | - | - | 230-590 EUR/t NO_X_^f^  (EPA; 2015) |
| Iron and steel production –  Low-NO_X_ burning combined with flue gas recirculation, selective catalytic reduction | - | - | - | 560-3,370 EUR/t NO_X_^f^  (EPA; 2015) |
| By-product coke manufacturing – selective non-catalytic reduction | - | - | - | 820 EUR/t NO_X_^f^  (EPA; 2015) |
| Petroleum refining (incl. gas-fired processes) – selective catalytic reduction) | - | - | - | 850-8,310 EUR/t NO_X_^f^  (EPA; 2015) |
| Coal cleaning – thermal drying and low NO_X_ burning | - | - | - | 1,020-1,490 EUR/t NO_X_^f^  (EPA; 2015) |
| Stationary diesel and dual-fuel combustion (incl. for electricity generation) – ignition retardation, selective catalytic reduction | - | - | - | 1,140-3,470 EUR/t NO_X_^f^  (EPA; 2015) |
| Incinerators –  selective non-catalytic reduction | - | - | - | 1,670 EUR/t NO_X_^f^  (EPA; 2015) |
| Process heaters (gas fired and others) – ultra-low NO_X_ burning, selective catalytic reduction | - | - | - | 2,030-2,400 EUR/t NO_X_^f^  (EPA; 2015) |
| Natural gas stationary combustion for electricity generation – adjusted air to fuel ration and retarded ignition | - | - | - | 2,130 EUR/t NO_X_^f^  (EPA; 2015) |
| Catalytic cracking, process heaters, coke ovens – flue gas recirculation, low-NO_X_ burning | - | - | - | 2,150-4,730 EUR/t NO_X_^f^  (EPA; 2015) |
| Boilers (Industrially-commercially-institutionally used, incl. coal and residual oil boilers) - low-NO_X_ burning combined with flue gas recirculation, selective catalytic and non-catalytic reduction | - | - | - | 2,190-3,140 EUR/t NO_X_^f^  (EPA; 2015) |
| Nitric acid production –  non-selective catalytic reduction | - | - | - | 2,430 EUR/t NO_X_^f^  (EPA; 2015) |
| Ammonia production –  selective catalytic reduction | - | - | - | 2,630 EUR/t NO_X_^f^  (EPA; 2015) |
| Glass manufacturing –  OXY firing | - | - | - | 2,820-6,800 EUR/t NO_X_^f^  (EPA; 2015) |
| Miscellaneous industrial processes – flue gas recirculation, low-NO_X_ burning | - | - | - | 3,660 EUR/t NO_X_^f^  (EPA; 2015) |
| Taconite ore processing – selective catalytic reduction | - | - | - | 5,860 EUR/t NO_X_^f^  (EPA; 2015) |

^a^ We uniformly assume an exchange rate of 1.10 USD/EUR.

^b^ We assume for the lower margin a cost reduction of 30% between 2012 and 2017.

^c^ We assume consumption and price of urea solution to be 1.5 l per 1,000 km and 1.10 EUR/l, respectively.

^d^ In the case of after-treatment technologies for light-duty vehicles, we assume technology levels necessary to comply with the Euro 6 emissions limits for light-duty vehicles.

^e^ Considering production costs only.

^f^ Rough estimate based on costs given by EPA (2015) in USD_2011_; assuming an exchange rate of 1.10 USD/EUR.

^c^ We assume here a consumption and price of urea solution of 1.6 l per 100 km and 0.55 EUR/l, respectively.

Table S10: Data sources and assumptions used for estimating the costs of mitigating PN emissions of road vehicles; data represent estimates that indicate general cost trends (Source: Zerfass, 2017)

| **Technology^c^** | **Costs** | **Lifetime** | **Efficacy** | **Emission mitigation costs** |
| --- | --- | --- | --- | --- |
| Light-duty vehicles -  Diesel particulate filter  (including installation by car manufacturer) | 150-350 EUR (estimate based on Possada Sanches, 2012)^a,b^ | 200,000 km (based on FG, 2017; Giechaskiel, 2017 | 95% PN removal efficacy (Giechaskiel, 2017) to a level of 6*10^11^ particles/km (based on Giechaskiel et al., 2015) | 7-23 EUR/10^17^ particles |
| Light-duty vehicles –  Diesel particulate filter  (retrofit by vehicle user) | 500-2000 EUR (own estimate based on DHZ, 2016; FG, 2017) | 200,000 km (based on FG, 2017; Giechaskiel, 2017 | 95% PN removal efficacy (Giechaskiel, 2017) to a level of 6*10^11^ particles/km (based on Giechaskiel et al., 2015) | 22-88 EUR/10^17^ particles |
| Light-duty vehicles –  Gasoline particulate filter | 67-158 EUR (Minjares and Posada Sanchez, 2011)^a,b,e^ | 200,000 km, assumption based on DPFs | 70% PN removal (Giechaskiel, 2017); engine out emissions of 1,4*10^12^ particles/km decreased to 5-6*10^11^ particles/km (Bischof et al., 2012) | 34-79 EUR/10^17^  particles |
| Heavy-duty vehicles –  Diesel particulate filter | 980-1,560 EUR (estimate of manufacturing costs for catalyzed DPFs based on Possada, et al., 2016)^d,e^ | 500,000 km based on Euro VI durability requirements (Delphi, 2017) | 95% PN removal efficacy (Giechaskiel, 2017) to a level of 4*10^11^ particles/km (based on Giechaskiel et al., 2016 and an energy use of 1 kWh/km) | 26-41 EUR/10^17^ particles |

^a^ We uniformly assume an exchange rate of 1.10 USD/EUR.

^b^ We assume for the lower margin a cost reduction of 30% between 2012 and 2017.

^c^ In the case of after-treatment technologies for vehicle emissions, we assume technology levels necessary to comply with the Euro 6 emissions limits for light-duty vehicles.

^d^ Considering production costs only.

^e^ Rough estimate based on costs given by EPA (2015) in USD_2011_; assuming an exchange rate of 1.10 USD/EUR.

Text Box 1: Specific price of electric cars in terms of battery capacity

Expressing the price of electric cars in terms of battery capacity [kWh], we find a decline in the mean specific price by 50% from 2,500 ± 1,500 EUR_2015_/kWh in 2010 to 1,240 ± 430 EUR_2015_/kWh in 2016. The experience curve analysis reveals a learning rate of 16 ± 2% (Figure 3), which differs from the 23 ± 2% observed in Figure 2a. As this finding suggests, the choice of motor power [kW] versus battery capacity [kWh] as functional unit indeed affects the result of the experience curve analysis despite the strong correlation between the two parameters.

Figure S1: Experience curve depicting the mean specific price of electric cars (BEVs) expressed per unit of battery capacity [kWh]; error intervals represent the standard deviation of price data

Text Box 2: Key uncertainties related to our experience curve analysis

1. We approximate production costs by vehicle price, thereby assuming constant profit margins. As suggested by the rapidly declining prices of electric cars between 2010 and 2014 and the price stagnation in the period afterward, profit margins may not have remained constant but instead declined after a larger number of manufacturers began selling electric cars and plug-in hybrids. If so, the learning rates estimated here may not remain constant but could decline in the future.

2. The technical characteristics of electric and plug-in hybrid cars have arguably been changing in the period of our analysis, most notably through the introduction of novel infotainment systems, driver’s assistance, and safety features. Such technological heterogeneity leads to a, supposedly small, underestimation of learning rates.

3. The identified learning rates are technology specific. That is, if the technology of electric and plug-in hybrid cars change in the future (e.g., through the introduction of wheel-hub motors, advanced semiconductors, or solid-state batteries), learning rates may change as well. Therefore, caution is needed when using our findings to forecast the prices and production costs of electric and plug-in hybrid cars in the long term.

4. As the battery capacity of individual electric and plug-in hybrid car models tends to increase over time (Zerfass, 2017), cumulative battery capacity may be a more reliable experience indicator than the number of vehicles sold. The latter indicator may, therefore, underestimate the accumulated experience and thus overestimate learning rates. Future research could seek to advance this point.

5. We calculate learning rates based on the yearly mean price and price differential of electric and plug-in hybrid cars and thus do not account for the variability of these parameters within individual years. Our choice ensures each year receives an equal weight in the experience curve analysis, but it fails to account for actual sales of individual models and it disguises uncertainty arising from the large variability in the price of electric and plug-in hybrid car models sold each year.

Text Box 3: Uncertainty in the comparison of emissions mitigation costs between electric and after-treatment systems in conventional cars

1. Figures S1 and S2 assume that after-treatment technologies decrease engine-out NO_X_ and particle number emissions to emission levels as they occur during normal vehicle use (see Zerfass, 2017) up to a vehicle age of 5 years or a mileage of 160,000 km as it is prescribed by regulatory durability requirements (EC, 2007). If after-treatment systems degrade (normally without being noticed by the vehicle user) within and after this period, the actual costs for mitigating tailpipe emissions through exhaust after-treatment might be substantially higher than suggested in Figures 8 and 9.

2. Figures S1 and S2 do not account for exposure to NO_X_ and particles. The proximity of vehicle use to high population density in cities implies that citizens are more likely exposed to vehicle emissions than to emissions from the energy and manufacturing sector; considering actual exposure when benchmarking the costs for emissions mitigation will improve the cost performance of technologies that mitigate the emissions of road vehicles as compared to those that mitigate the emissions of the energy and manufacturing sector.

3. The estimates in Figure 6 (main text) depict the costs of decreasing NO_X_ and PN emissions through electric cars and plug-in hybrids *beyond* the current emission levels of conventional cars; by contrast, the cost estimates in Figures 7 and 8 for after-treatment technologies depict the costs of decreasing emissions from engine-out levels *to* the current emission levels of conventional cars. The costs estimates for electric cars and plug-in hybrids therefore represent *marginal* costs; the cost estimates for after-treatment systems represent *average* costs – the latter could increase if tailpipe emissions are to be decreased in the future to more stringent emission limits.

Text Box 4: Uncertainty related to on-road NO_X_ and PN emission factors

On-road measurements of NO_X_ emissions from plug-in hybrid cars are still scarce; the same applies to the measurement of particle number emissions from both diesel and gasoline cars. Hammer et al. (2015) suggests slightly higher particle number emissions for diesel plug-in hybrids than for conventional diesel cars. If one considers that plug-in hybrid cars can be driven at least temporarily by an electric motor and thus without tailpipe emissions, there is *a priori* no reason to assume higher particle number emissions for Euro 6 plug-in hybrids than for conventional diesel cars. However, reality is more complex. Vehicle manufacturers face cost constraints, which could lead to the installation of smaller catalysts and particulate filters or a decreased dosing of urea solution in plug-in hybrids and in turn to overall elevated NO_X_ and particle emissions. Such considerations may justify the NO_X_ and particle number emission factors chosen here but also ask to substantiate these by further research. Probabilistic modeling could accommodate a range of likely emission factors and operating conditions, specifically those relevant for urban driving.
